# Prediction of Oral Healthcare Services Utilization in Saudi Arabia

**DOI:** 10.7759/cureus.87062

**Published:** 2025-06-30

**Authors:** Yaser A Alsahafi

**Affiliations:** 1 Department Oral Health and Epidemiology, Faculty of Dentistry, Qassim University, Buraydah, SAU

**Keywords:** district health information system, healthcare system, healthcare system in saudi arabia, health policy making, health service utilisation, health system research, oral health care

## Abstract

Background

The Saudi Ministry of Health is the major oral healthcare provider in Saudi Arabia. The ministry releases annual reports on oral healthcare delivery and usage indicators in the public sector for the 13 administration provinces. However, the Saudi Ministry of Health and population census data are not synchronized to estimate the utilization of regional oral healthcare. This study aims to find the predictors of oral healthcare services utilization in Saudi Arabia.

Methods

Oral health data for the 13 Saudi provinces were collected. Healthcare parameters and population data were obtained from the Saudi Ministry of Health and the Saudi Central Department of Statistics and Information, respectively. To study oral healthcare utilization, number of dental visits, number of dentists, dental treatment needs, caries prevalence, and province population were used as indicators. Oral healthcare utilization was predicted by a multiple linear regression using the number of dental visits, number of dentists, treatment needs, caries prevalence, and province population.

Results

The regional predication of oral health utilization was influenced mainly by population at the province level and number of dentists in the province. Although caries prevalence and treatment needs have a great impact on the prediction of oral healthcare utilization, their association with utilization was not statistically significant.

Conclusion

The main predictors of oral healthcare utilization at the province level in Saudi Arabia are province population and number of dentists. Caries prevalence and treatment needs are valuable modifiers for the prediction. For future research, the availability of sub-province data is needed to enhance the ability to predict oral healthcare utilization.

## Introduction

In many countries, the prevalence of dental caries ranges between 60% and 90% in school-age children [1]. In Saudi Arabia, the Ministry of Health reported a national caries prevalence of 96% [2]. The US Surgeon General reported, in 2000, that dental caries was the most common childhood disease and more than twice that of asthma and hay fever [3]. Dental caries severity is measured by a DMFT (Decayed, Missing, and Filled Teeth) index. Mean DMFT is used to describe the severity of caries at the community level [4]. Globally, DMFT has been declining for the past 30 years. Mean DMFT dropped from 2.43 in 1980 to 1.67 in 2011 among developed countries. Saudi Arabia is experiencing a rise in the mean DMFT to 5.9 in 2002, from 2 in 1979. Saudi DMFT is the highest among countries included in the WHO oral health database [1].

The population determinants of oral health utilization have been assessed in countries of various development levels. The spatial distribution of dental caries to social deprivation has been evaluated in a school-based study of 25 schools in districts in Brazil [5]. This study provided evidence that DMFT and family income percentage are good determinants for district-level caries distribution. The factors that influence the frequency of and reason for dental visits in teenagers in Chile were investigated [6]. This study demonstrated that oral behavior and socioeconomic status are important aspects of dental healthcare utilization behavior and can help improve access to healthcare in that population. The predictors of poor working adults’ use of oral health services in Canada were investigated in a cross-sectional study [7]. The investigation revealed that perceived dental needs, having more than 21 teeth, and the ability to pay for services could be predictors for oral healthcare utilization. The predictors of future utilization of prosthetic dental treatment were investigated in a longitudinal study in Brazil [8]. The study suggested that the level of dental caries, level of mother's education during childhood, and oral health awareness are the main predictors of the future need for dental care [8]. A review of literature on recent changes in determinants of oral health services at the community level focused on literature on the transformation in dental disease patterns, belief in diagnostics and treatment, and the amount of clinical effort [9]. The study revealed that previously used determinants are only valid after considering population measures and changes in oral health practices into consideration. The determination of dental treatment demand by level and severity of dental caries was evaluated among the population aged 45 years and older in Kaunas city, Lithuania [10]. The study suggested that behaviors and perceptions are significant predictors of dental care demand. The ability to anticipate dental practice transformation based on current circumstances was evaluated by Zubiene et al. This suggested that health equity, demographics, and societal factors could be predictors of potential changes in dental care practice [11]. Javali et al. evaluated the expenditure on oral healthcare using the predicted distance to providers. Their findings suggested that, after controlling for other health determinants, the spatial location of dentists was a significant predictor of expenditure on oral healthcare [12].

In Saudi Arabia, Al Dosari et al. evaluated the concentration of water fluoride intensity and its relation to dental caries distribution among children in selected provinces [13]. The study suggests a lack of any correlation between the prevalence of dental caries and the intensity of water fluoride [13]. In addition, the investigation provides an insight into Saudi Arabia as a study area. The average caries prevalence at the province level in Saudi Arabia was almost 84%, in the most recent study, with 60% unmet dental treatment needs [14]. Among the Saudi Arabian population, the national average caries prevalence in Saudi Arabia is 93% [15]. The average dental treatment needs was reported to be 59.32% in Saudi Arabia, which has 13 provinces with a total population of almost 32 million [16]. The Saudi Ministry of Health (MOH) reported the average dentist ratio is 4.11 dentists per 10,000 population [17,18]. According to the previous literature, the high caries level and high dentist ratio could have an impact on oral healthcare utilization. However, the predictors of oral healthcare utilization in Saudi Arabia have not been investigated. The aim of the current study is to determine the predictors of oral healthcare utilization in Saudi Arabia. The study is secondary data analysis based on public records and findings from published literature.

## Materials and methods

Study sample

The 13 provinces of Saudi Arabia were the target population of this study. This study design is a cross-sectional observational analytic study. Dental resources data and dental caries measures data from the provinces were collected for analysis.

Study data sources and analysis

This current study is a secondary data analysis of published data. The utilization of health services is determined by demand and supply. In this study, oral healthcare utilization, caries prevalence, number of dental visits, and the population of dental treatment needs were used as proxies for the demand for oral health services. The number of dentists and oral healthcare facilities and their locations were used as proxies for oral health services supply. Both demand and supply were evaluated in Saudi Arabia using province-level variables.

Data Determination and Acquisition

To investigate oral healthcare utilization, various public recodes were used to obtain demand and supply variables at the province level. The dentist-to-population ratio was used as a proxy indicator for access to oral healthcare services. Data sources for all variables are listed in Table 1. The number of dental visits and the number of dentists were obtained from the Saudi Ministry of Health.

**Table 1:** Data sources for Saudi population and health indicators

Data	Description	Source
Saudi Arabian population	Table: population by province	Central Department of Statistics and Information (CDSI), Ministry of Economy and Planning, Saudi Arabia
Health care measures	Tables: Oral health work force and no. of dental visits by province	Ministry of Health (MOH), Saudi Arabia
Dental caries measures	Tables: Caries prevalence, treatment needs by province	National Campaign to Prevent Dental Caries (NCPDC), Saudi Arabia

Data Management

The healthcare facilities data, namely number of dentists and number of dental visits, were available in governmental healthcare facilities. Data from private healthcare facilities were excluded from this study because province-level data for non-governmental healthcare facilities were not publicly available.

Statistical Analysis

Descriptive analysis was performed to assess variable distribution and parameters on province-level oral health and healthcare. Bivariate analysis was performed to assess the association between oral health and population parameters. Multiple linear regression was conducted to evaluate the predictors of oral healthcare utilization. The number of dental visits was the dependent variable, and the independent variables were population size, caries prevalence, dental treatment needs, and number of dentists. Because multiple linear regression assumes linearity, all variables were examined for normality. Caries prevalence and number of dental visits were not normally distributed, but proximity to normality increased when these variables were transformed to their log value. The assessment of the number of dentists and oral health treatment needs revealed their proximity to normality. The findings from normality analysis were taken into consideration during model building. Multiple linear regression analysis was performed using the IBM SPSS Statistics (version 25, IBM Corp, Armonk, NY).

## Results

Descriptive analysis

Central tendency, maximum, and minimum findings for all the supply and demand variables were calculated (Table 2). We observed normal distributions for the population per dentist ratio in provinces, caries prevalence, and dental treatment needs. The average dentist-to-population ratio was 1.3 dentists per 10,000 population. The number of dentists and number of dental visits showed a right-skewed distribution. This finding suggests notable disparities among provinces, with some having substantially more dentists and dental visits than others.

**Table 2:** descriptive analysis of oral health indicators. Population>10 years old: population over 10 years old; SD: standard deviation, Min.: minimum, Max.: maximum.

Variable	Mean	Median	SD	Min. – Max.
Caries prevalence	82.07%	81.9%	5.8%	71.4-89.4%
No. of dental visits	42926	21604	4688.7	6882-150335
No. of dentists	201.09	103	173.4	54-577
Dental treatments	58.77 %	62.6%	10.3%	38.4%-70.6%
Population>10 years old	1,551,650.27	735,682	1,787,613.3	294,896-6,097,077
Population per dentist	7494.8	7266.7	2510.9	3476.2-10723.37

Providence-level oral health indicators

**Figure 1:**
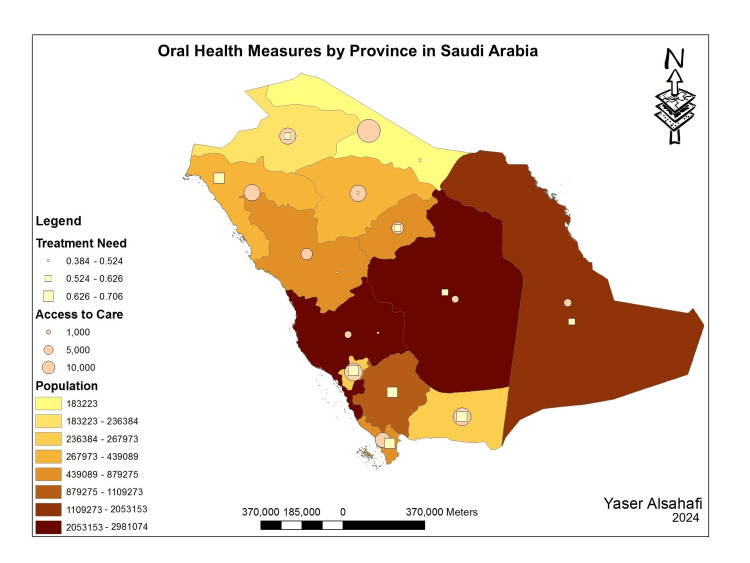
Population, treatment need, and access to care by province in Saudi Arabia Spatial presentation of the data was creating using ArcGIS 10.1 (ESRI, 2012). ESRI 2012. ArcGIS Desktop for Windows (Version 10.1) (computer software). Redlands, CA: Environmental Systems Research Institute.

The province-level analysis of the oral health indicators revealed that access to oral healthcare was inversely related to province's treatment needs (Figure 1).

**Figure 2:**
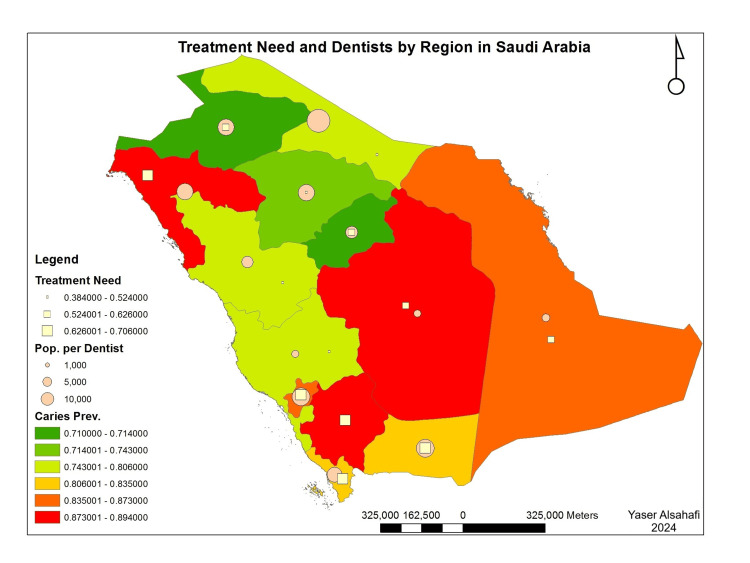
Treatment needs and number of dentists by province Spatial presentation of the data was creating using ArcGIS 10.1 (ESRI, 2012). ESRI 2012. ArcGIS Desktop for Windows (Version 10.1) (computer software). Redlands, CA: Environmental Systems Research Institute.

**Figure 3:**
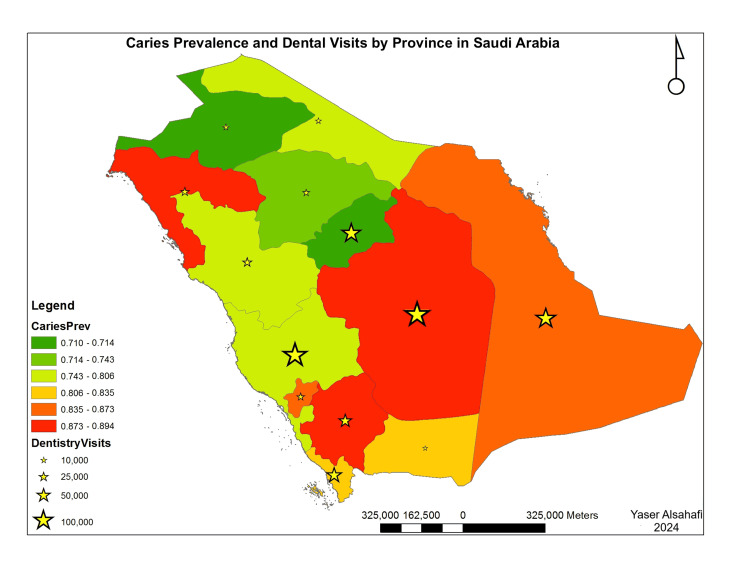
Caries prevalence with no. of dental visits by province Spatial presentation of the data was creating using ArcGIS 10.1 (ESRI, 2012). ESRI 2012. ArcGIS Desktop for Windows (Version 10.1) (computer software). Redlands, CA: Environmental Systems Research Institute.

However, this pattern was only true for provinces with population extremes. In addition, the number of dental visits was inversely correlated to access to oral healthcare (Figure 2). However, these associations were true regardless of the prevalence of caries at the province level. Moreover, provinces with extremely high caries prevalence had a high number of dental visits. Still, this association was not true in areas with very low caries prevalence (Figure 3). The spatial presentation of the indicators revealed a potential pattern in which access to oral healthcare was inversely related to province treatment needs (Figures 2, 3). However, this pattern was only true for provinces with population extremes.

Multiple linear regression (MLR)

The multiple linear regression was built to fit (satisfy) this equation:

$Y = \beta_0 + \beta_1 \cdot (\text{province population}) + \beta_2 \cdot (\text{caries prevalence}) + \beta_3 \cdot (\text{dental need}) + \beta_4 \cdot (\text{no. of dentists}) + \varepsilon$

Stepwise model selection (0.05 entry, 0.1 leave) was used to build the model. The best-fit model had an R^2^ of 0.99. The model satisfied the following equation:

$\text{No. of dental visits} = 38629.913 + 0.1 \cdot (\text{pop.} > 10) - 232.672 \cdot (\text{No. of Dentists}) - 97124.14 \cdot (\text{Caries Prevalence}) + 76727.967 \cdot (\text{Treatment Needs})$

The interpretation of the model is as follows: for every one million increases in the population, the number of oral health visits will increase by 0.1, given that all other predictors were unchanged. This model explained 99% of the variability of predicting the number of dental visits (Table 3).

**Table 3:** Oral health utilization predictors for the best fit prediction model Std. Error: Standard error, *p*-value: significance level

Model	Unstandardized Coefficients		p-value	95.0% Confidence Interval	
	B	Std. Error		Lower Bound	Upper Bound
(Constant)	38629.913	37650.252	00.344	-53496.935	130756.761
Population	00.100	00.016	00.001	00.060	00.140
No. of dentists	-232.627	81.057	00.028	- 430.968	-34.287
Caries prevalence	-97124.14	62405.045	00.171	-249823.79	55575.504
Treatment needs	76727.967	33991.834	00.065	-6447.053	159902.988

The association between the number of dentists and the number of oral healthcare visits demonstrates a statistically significant relationship. The number of visits would decrease by 232 dental visits for each decrease in the oral health workforce by one dentist. Although treatment needs demonstrate borderline statistical significance, the 95% confidence interval indicates low precision in the predictability.

## Discussion

In this study, data were mined from the Saudi Arabian databases for oral health service, census, regional disease distribution to ascertain the common predictors of oral healthcare utilization within the Saudi Arabian healthcare system. There is an extremely high national caries prevalence in Saudi Arabia [2] and DMFT index is high in Saudi Arabia among the countries in the WHO oral health database [1]. The demand predictors evaluated in the current study were based on previous suggested oral healthcare utilization predictors in the literature for comparable and dissimilar healthcare systems around the world [5-11].

The data used for the prediction model were based on the most recent country-wide studies or official reports. Despite the availability of recent studies, none of them have national-level data, nor do they cover all age groups. In addition, the caries prevalence and dental workforce used in the current study are up-to-date and provided by healthcare authorities in Saudi Arabia [14-18].

The study revealed multiple correlations between oral health parameters and healthcare parameters. The directions of those correlations were equivalent to the literature findings [5,6,9]. However, previous studies were able to investigate specific predictors for oral healthcare utilization at the sub-province level. In our study, data on sub-province level (such as governorate or city level) were not available for investigation. The potential source for the correlation in our study is the supply of oral healthcare system and demand for oral healthcare utilization. Caries prevalence is the most likely driver for demanding oral healthcare in terms of dental visits. Our study also observed a supply and demand correlations were different in provinces with extremely high number of dental visits. Further analysis should be conducted to find the underlying causes using governate or city-level data.

The prediction model evaluated in this study for estimating demand for oral healthcare incorporated a future change in the population of the province. The model analysis demonstrated the ability to explain 99% of the prediction variability. The model finding is consistent with the current literature review, which suggested that the utilization of oral health services can be significantly predicted by the regional location of dentists [12].

The findings revealed overall scarcity of dentists, especially in provinces with the most needs, despite the reported overall high dentist-to-population in Saudi Arabia [17,18]. Dentists’ concentration in major provinces and major cities could be due to the availability of better work opportunities and social environment resources such as schools, entertainment, continuous education, and financial stability. The health equity was also revealed in the variation in caries prevalence between provinces. Although overall caries prevalence is high in Saudi Arabia [14], our findings revealed a higher prevalence in two of the highest populated provinces, namely, Riyadh and Asir [19]. This observation is possibly due to a high number of construction projects and service industries that attract a population of low-income workers with low education, a combination that was previously associated with lower oral health literacy [8]. The inverse correlation observed between treatment need and access to oral healthcare could be justified by the recently published high unmet oral treatment needs (60%) among school children [14] and the previously reported high need for care (59.3%) for all ages [15]. However, the scarcity of data on socioeconomic status (SES) factors in the province hinders the assessment of prediction based on these health indicators.

Study limitations

The findings from this analysis provide essential insight into the predictors of oral health utilization in Saudi Arabia at the province level. However, various limitations, primarily related to data availability, ought to be discussed to contextualize and guide further research.

Primarily, province-level data were utilized in this study and such data limits the analysis granularity. The lack of granular province-level data, such as governorate or city data, restricts the ability to capture variation of oral health care utilization at the inter-province level. Significant disparities might exist within provinces, such as differences between rural and urban areas, that could not be detected in this study. A deeper analysis could have been possible if sub-province data were available and such analysis would have provided a better understanding of local oral health utilization patterns and healthcare needs, allowing for more precise policy recommendations.

Another significant limitation is lack of private healthcare facilities data. In Saudi Arabia, governmental and private providers deliver oral healthcare services. This study focused on only data from the public health sector, which may have underrepresented the true demand for dental care. The utilization of oral healthcare in the private sector might be preferable for higher-income population, which could potentially skew the analysis of healthcare utilization across different socio-demographic groups. To reveal a more comprehensive view of the overall oral healthcare utilization, future studies should include private sector data. In addition, private sector data may also reveal important trends in service utilization.

Data on income level, education, employment status, and other SES indicators were not available for analysis. Utilizing healthcare services and accessing them are determined by SES indicators. The assessment of the influence of social and economic inequalities on oral healthcare utilization was diminished by the exclusion of SES. Future models that integrate SES data at the province level would lead a robust assessment of social and economic determinants of health. Furthermore, vulnerable population could be identified by models incorporating SES, which could support planning targeted interventions.

Finally, a large proportion (99%) of oral healthcare utilization variability was explained by the multiple linear regression model. However, a limited prediction precision was observed for the treatment need and some other predictors. More precise data for predictors with large confidence interval and borderline statistical significance would improve prediction model accuracy in future studies. Moreover, the inference of causality between predictors and oral healthcare utilization is not possible using the current cross-sectional study design, which was suitable for the current exploratory study using available data. Future studies using a longitudinal design is mandatory for capturing temporal change in healthcare utilization.

## Conclusions

The oral healthcare utilization at the province level in Saudi Arabia can be predicted by caries prevalence, treatment needs, dentist-to-population ratio, and number of dentists. The current study developed a model for estimating the future capacity of the oral healthcare system at the providence level. Further research could investigate the baseline health indicators at the governate or city level to build a model for forecasting a province-specific dental workforce volume needed.
